# Genome-Wide Discovery of G-Quadruplexes in Wheat: Distribution and Putative Functional Roles

**DOI:** 10.1534/g3.120.401288

**Published:** 2020-04-15

**Authors:** H. Busra Cagirici, Taner Z. Sen

**Affiliations:** Western Regional Research Center, Crop Improvement and Genetics Research Unit, United States Department of Agriculture—Agricultural Research Service, 800 Buchanan St., Albany, CA 94710

**Keywords:** Genome analysis, wheat, G-quadruplexes, plants

## Abstract

G-quadruplexes are nucleic acid secondary structures formed by a stack of square planar G-quartets. G-quadruplexes were implicated in many biological functions including telomere maintenance, replication, transcription, and translation, in many species including humans and plants. For wheat, however, though it is one of the world’s most important staple food, no G-quadruplex studies have been reported to date. Here, we computationally identify putative G4 structures (G4s) in wheat genome for the first time and compare its distribution across the genome against five other genomes (human, maize, Arabidopsis, rice, and sorghum). We identified close to 1 million G4 motifs with a density of 76 G4s/Mb across the whole genome and 93 G4s/Mb over genic regions. Remarkably, G4s were enriched around three regions, two located on the antisense and one on the sense strand at the following positions: 1) the transcription start site (TSS) (antisense), 2) the first coding domain sequence (CDS) (antisense), and 3) the start codon (sense). Functional enrichment analysis revealed that the gene models containing G4 motifs within these peaks were associated with specific gene ontology (GO) terms, such as developmental process, localization, and cellular component organization or biogenesis. We investigated genes encoding MADS-box transcription factors and showed examples of G4 motifs within critical regulatory regions in the VRN-1 genes in wheat. Furthermore, comparison with other plants showed that monocots share a similar distribution of G4s, but Arabidopsis shows a unique G4 distribution. Our study shows for the first time the prevalence and possible functional roles of G4s in wheat.

Molecular functions and biological processes are highly dependent on the nucleic acid structure of a genome (Ding *et al.* 2014). Both DNA and RNA sequences can form several secondary structures, such as loops, hairpins, duplexes, triplexes, and quadruplexes, to regulate diverse biological mechanisms (Wan *et al.* 2011; Leppek *et al.* 2018). G-quadruplexes (G4) are four-stranded nucleic acid structures formed within guanine-rich sequences. Closely spaced guanine (G) bases are able to form a square planar structure of G-quartets where a stack of G-quartets forms G4s (Figure 1A). There are several patterns suggested for G4 structures with varying number of consecutive G bases, called a G stem, connected by relatively flexible regions of nucleotides called a loop. The annotation of the G4 patterns is based on the number of bases in a G stem and the loop lengths in between (Hazel *et al.* 2004). For example, two G bases forming a stem followed by a short loop of 1 to 3 bases were categorized as G_2_L_1-3_ whereas G_5_L_1-7_ denotes five consecutive Gs followed by a long loop (Figure 1B) (Yadav *et al.* 2017). *In vitro* evidence demonstrated the G_3+_L_1-7_ pattern to form the most stable G4 structure (Bugaut and Balasubramanian 2008; Mullen *et al.* 2010) (Figure 1A and 1C).

**Figure 1:**
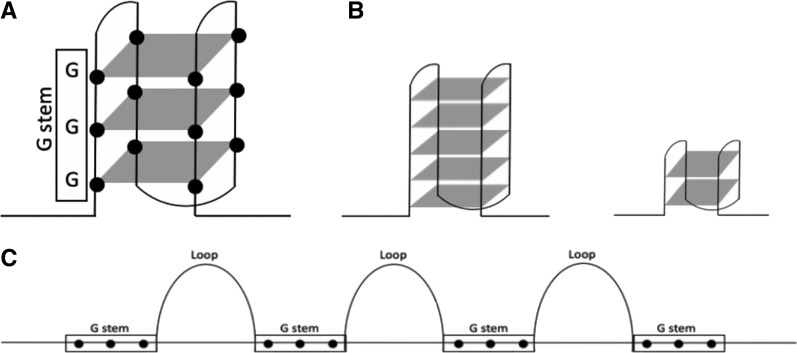
G-quadruplex folding. G bases are shown as black dots. Three consecutive G bases, called G-stem, was shown in boxes. G-quartets were represented as gray planar squares. A stack of three G-quartets on top of one another forms a G-quadruplex structure. (A) Representation of the shortest stable G4 pattern G_3_L_1-7_. The subscript under G represents the number of guanine bases in the G stem, and the subscript under L is the number of bases in the loop connecting G stems. In the specific case of G_3_L_1-7_, 1-7 denotes the number of bases forming loops are between one and seven. (B) A couple of examples for G-quadruplex patterns based on varying numbers of G-quartets were shown: G_5_L_1-7_ on the left and G_2_L_1-3_ on the right. (C) Linear sequence representation of potential G-quadruplex motifs, such as the one shown in (A).

Genome-wide in silico studies in human and plants revealed that G4 structures are highly enriched in certain loci, such as telomeres (Moye *et al.* 2015), promoters (Huppert and Balasubramanian 2007), ribosomal DNA, translation start sites (TSSs) (Takahashi *et al.*, 2012) and 5′ UTRs (Huppert *et al.* 2008). For genes containing G4 structures, special helicases are required to unwind the G4 structures to continue replication or transcription (Huber *et al.* 2006; Mendoza *et al.* 2016). It was argued that these G4 structures can impede polymerase progression, affecting several biological functions. *In vitro* and *in vivo* studies have indeed evinced the important roles of G4 structures in various biological processes, including gene regulation, cell growth, and development (Juranek and Paeschke 2012; Clark *et al.* 2012; Yuan *et al.* 2013).

Some biological functions of G4 structures have been verified using small molecules which stabilize G4 formation through binding (McLuckie *et al.* 2013; Neidle 2017). *In vitro* evidence showed that stabilization of G4 structures by ligands is associated with the translation inhibition of downstream genes (Bugaut *et al.* 2010; Halder *et al.* 2011). In addition, Ito *et al.* demonstrated the inhibition of translation in living cells using a short G-rich RNA that form an intermolecular G-quadruplex in the 5′ UTR region of the target gene (Ito *et al.* 2011). Overall, these studies demonstrated regulatory and functional importance of G4 structures.

Genome wide analyses of G4 structures have been reported for many species including humans, mouse, and fungi. On the other hand, only a limited number of studies is available for plant species. *In vitro* studies confirmed the formation of G4 structures and demonstrated the effect of G4 structures in stalling DNA replication in plant genomes like *Arabidopsis thaliana*, *Brachypodium distachyon*, and *Oryza sativa* by using circular dichroism spectroscopy and gel electrophoresis (Garg *et al.* 2016). Functional annotation of these genes containing G4 structures was associated with development, transcription regulation, and protein folding in monocot plant species (Garg *et al.* 2016). However, no study has showed the distribution and composition of G4 structures in wheat species to date.

Wheat (*Triticum aestivum*) is one of most important staple food sources for human consumption, its production rate ranks second over global crop production (FAO 2019). The recent availability of the bread wheat reference sequence (Appels *et al.* 2018) has paved the way for the elucidation of genomic elements and their respective functions in wheat species (Adamski *et al.* 2020). In the present study, we investigated for the first time the G4 content of a wheat genome using the IWGSC RefSeq v1.0 assembly. We have revealed that G4 motifs were enriched on genic hotspots on the sense and antisense strands. Analysis of the functional annotation of gene models containing G4 motifs in the close proximity of start codons revealed that these G4s are associated with several important biological processes in wheat. Further, we compared the G4 contents in several mammalian and plant genomes with respect to the bread wheat genome.

## Materials and Methods

### Whole genome sequence and annotation datasets

The wheat reference genome assembly (IWGSC RefSeq v1.0) were obtained from wheat-URGI database together with high confidence gene annotation data (v1.1) (Appels *et al.* 2018). Unassigned scaffolds that were merged to form an unmapped pseudomolecule (chrUn) and the genes mapped to it removed in our analysis. For comparison purposes, we analyzed genomes of human and four plant species including *Arabidopsis thaliana*, *Zea mays*, *Oryza sativa japonica* and *Sorghum bicolor*. The whole genome sequences and annotations were obtained from the TIGR version 7 for *Oryza sativa japonica* (Ouyang *et al.* 2007). Data for the remaining species were downloaded from NCBI genome database (Agarwala *et al.* 2018): human (*Homo sapiens*, accession number: GCF_000001405.39), Arabidopsis (*Arabidopsis thaliana*, *GCF_000001735.4)*, maize *(Zea mays*, *GCF_000005005.2)* and sorghum *(Sorghum bicolor*, *GCF_000003195.3)*.

### In silico identification of G4 motifs

The most stable G4 motifs was reported as the G_3+_L_1-7_ pattern (Huppert and Balasubramanian 2005; Bugaut and Balasubramanian 2008; Mullen *et al.* 2010). The G_3+_L_1-7_ pattern was defined as {G_3+_L_1-7_}_3+_G_3+_ where G indicates guanine, L indicates any base in the loops, 1-7 indicates any number between one and seven and 3+ indicates 3 times or more (Mullen *et al.* 2010). For the G4 motifs on the antisense strand, we used the C_3+_L_1-7_ motif (reverse of the G_3+_L_1-7_ pattern). Putative G4 structures were extracted from both strands of genomes by searching any nonoverlapping motifs with the G_3+_L_1-7_ pattern using custom python scripts. Since the N bases can be any base, we accept N bases only if they are on the loop sites.

### G4 structure distribution analysis

Target regions were extracted based on gene annotations using custom python scripts. G4 structures on gene structural elements and other regions were retrieved using the intersect function of bedtools v2.29.0 (Quinlan and Hall 2010). G4 densities were calculated as total number of G4 structures per total length of the surveyed region. Positional G4 frequencies were calculated as the number of G4 elements at a given position, normalized by the number of genes.

### Functional analysis of G4 containing genes

Gene Ontology (GO) annotations for high confidence gene models were extracted from the IWGSC functional annotation file (iwgsc_refseqv1.0_FunctionalAnnotation_v1__HCgenes_v1.0.TAB), which was retrieved from wheat-URGI database. Suggested by the README file provided by IWGSC, transcript IDs with the same “6 digits” in v1.0 and v1.1 gene annotations correspond to the same genes. For example, ‘TraesCS1B01G365900.1’ corresponds to ‘TraesCS1B02G365900.1’ (Adamski *et al.* 2020). We retrieved the functional annotation of v1.1 gene models from the functional annotation of the corresponding genes in v1.0 gene models. Representative transcript for each gene model retrieved based on when the value of the “is_repr” column is one, when appropriate.

GO enrichment of genes was conducted using the BiNGO plugin for the Cytoscape (v3.7.2) visualization tool (Maere *et al.* 2005) and default statistical parameters were used for the hypergeometrical statistical test along with a Benjamini and Hochberg false discovery rate (FDR) correction at a significance level of 0.05. Comparison of two or more enrichment results were compared using WEGO v2 web tool (Ye *et al.* 2018).

### Data availability

The G4 sequences are publicly available at GrainGenes (Blake *et al.* 2019). The datasets are represented as a track in the IWGSC Chinese Spring genome browser at GrainGenes (https://wheat.pw.usda.gov/jb/?data=/ggds/whe-iwgsc2018). File S1 contains bed file for the G4 motifs identified in wheat reference genome v1.0. File S2 contains functional annotation of the genes containing G4 motifs within gene body and 1,000 bp upstream of high confidence gene models. File S3 contains functional annotation of the genes containing G4 motifs within the G4 peak regions. Figure S1 shows the distribution of G4s over the 10 kb regions centered at the start codon. Figure S2 shows the number of wheat homeologs containing G4 motifs. Figure S3 shows the G4 motif distribution for both high confidence genes and transcripts. Figure S4 shows GO terms that are significantly different between the enrichment analysis at the gene and transcript levels. Figure S5 shows the number of genes containing G4 motifs within three peak regions. Supplemental material available at figshare: https://doi.org/10.25387/g3.11348069.

## Results and Discussion

### Genome-wide discovery of wheat G4 motifs at the genome and subgenome levels

The bread wheat reference genome and high-confidence annotation data were obtained from the International Wheat Genome Sequencing Consortium (IWGSC) (Appels *et al.* 2018) as described in the *Methods* section. Using custom python scripts, we screened the wheat reference genome (v1.0) for G4 motifs as sequences containing at least three runs of G stems separated by loops of one to seven bases of any nucleotides, a pattern also referred as G_3+_L_1-7_, as the most stable G4 structure (Bugaut and Balasubramanian 2008). We identified more than 1,071,813 G4 motif instances with an average length of 28 bp (27.8) across chromosomes (Table 1). The sequences and genomic locations of predicted G4 structures (G4s) were provided in the File S1 and are available through the GrainGenes database (Blake *et al.* 2019). Our results showed that the G4 densities calculated over the 21 chromosomes only show small variations such that average G4 density was 76 G4 motifs per Mb with a variance of 9 (Table 1, Figure S1B).

**Table 1:** Distribution of predicted G4 structures (G4s) in wheat genome. Genic regions are composed of 5′ UTR, exon, intron, and 3′ UTR regions of high confidence gene models. G4 density indicates total number of G4 motifs per bp. Chromosomes which possess the highest and lowest G4 densities were highlighted with italic bold letters

			Whole genome		Genic regions	
Chromosome	Chromosome length	Average size of G4 motifs	# of G4 motifs	G4 density (x10^6^)	# of G4 motifs	G4 density (x10^6^)
1A	594,102,056	27.8	45,245	76	1,524	98
1B	689,851,870	27.5	52,963	77	1,617	96
1D	495,453,186	27.9	39,576	80	1,498	96
2A	780,798,557	27.8	58,308	75	1,875	93
***2B***	***801,256,715***	***27.6***	***58,814***	***73***	***1,837***	***85***
2D	651,852,609	27.9	51,315	79	1,886	94
3A	750,843,639	27.9	56,277	75	1,747	96
3B	830,829,764	27.6	61,460	74	1,863	90
3D	615,552,423	27.9	48,560	79	1,729	93
4A	7445,88,157	27.7	54,977	74	1,593	91
***4B***	***673,617,499***	***27.6***	***48,403***	***72***	***1,374***	***94***
4D	509,857,067	27.9	38,527	76	1,221	92
5A	709,773,743	27.9	54,601	77	1,751	95
5B	713,149,757	27.7	52,838	74	1,860	93
***5D***	***566,080,677***	***27.5***	***47,830***	***84***	***1,776***	***94***
6A	618,079,260	27.9	45,641	74	1,371	96
6B	720,988,478	27.6	54,982	76	1,556	92
***6D***	***473,592,718***	***27.8***	***38,427***	***81***	***1,259***	***88***
7A	736,706,236	28.2	56,095	76	1,729	92
7B	750,620,385	27.8	55,745	74	1,611	91
7D	638,686,055	27.9	51,229	80	1,693	93
**Overall**	**14,066,280,851**	**27.8**	**1,071,813**	**76**	34,370	**93**

**Figure 2:**
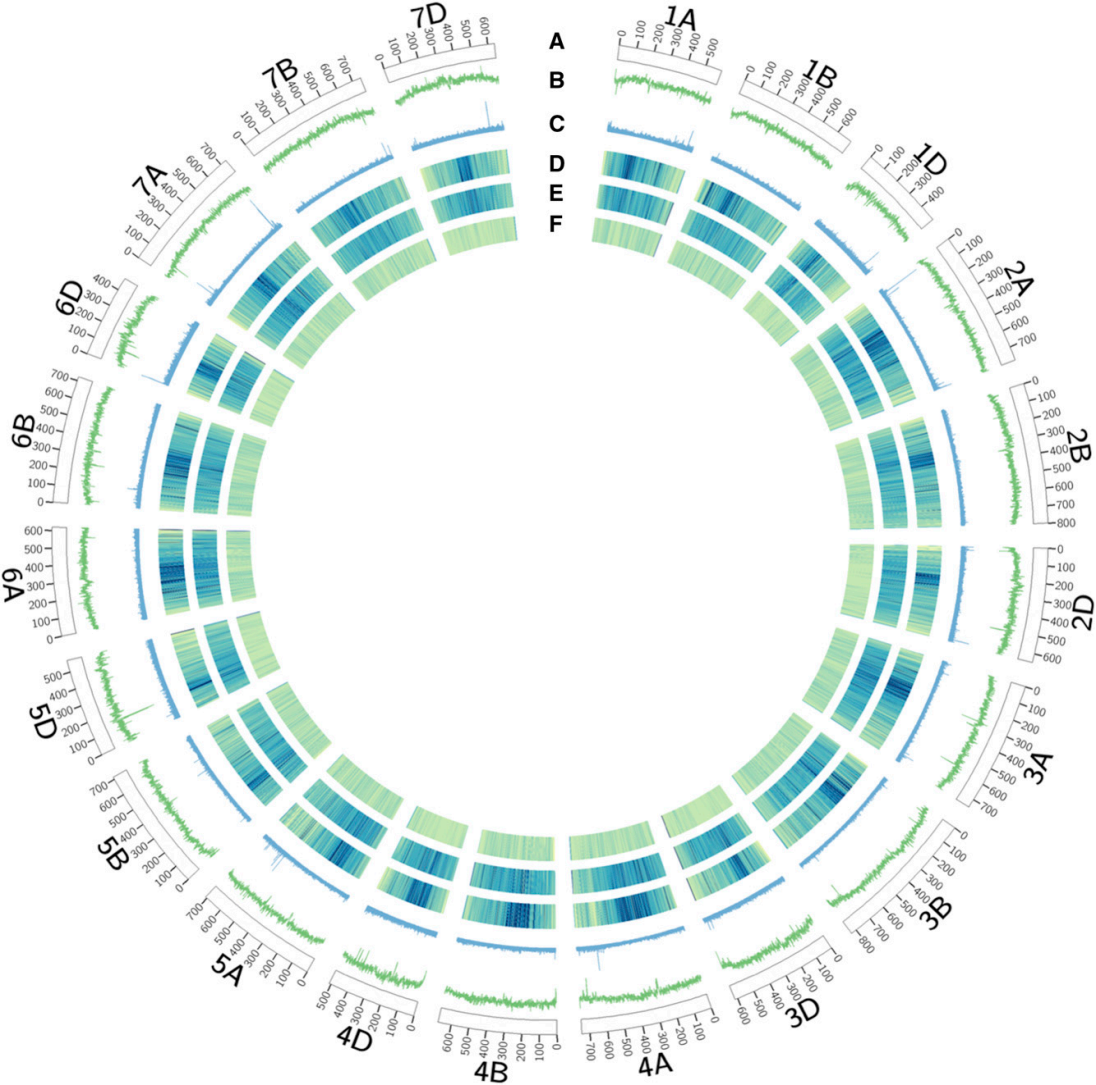
Circos diagram showing G4 motif density across wheat chromosomes in relation to the GC%, gene, and repeat content. From outside to inside: (a) ideogram of pseudomolecules (in Mb), (b) line graph of GC distribution as percentage to total number of bases, (c) line graph of G4 distribution as percentage of bases for G4 motifs to total number of bases, (d) histogram of high confidence gene density, (e) histogram of low confidence gene density, and (f) histogram of transposable element density. Histograms were plotted using the color palette; bugnyl-9-seq-rev. Highest values are yellow and the lowest values are blue.

In previous studies, the number of G4 structures were shown to be correlated with the GC content of the regions indicating an expected elevation in the number of G4 structures in the GC-rich genic regions (Mullen *et al.* 2010; Garg *et al.* 2016). To investigate the correlation among G4 content, GC percentage, and gene content, we prepared a Circos plot (Krzywinski *et al.* 2009) dividing individual wheat chromosomes into 1,000 bins. Figure 2 illustrates the G4 motif distribution across 21 wheat chromosomes, together with the GC percentage, high confidence gene models, low confidence gene models, and transposable elements. Our results show that G4 motifs are distributed throughout the chromosome; yet, are enriched mostly at chromosome ends. The chromosome ends containing telomeres are well-known regions for G4 structure formation (Neidle 2010; Jansson *et al.* 2019; Wu *et al.* 2020). Other than chromosomal end points, some of the G4 motif enriched regions were the regions enriched for high confidence gene models, such as ∼500-600 Mb regions on the chromosomes 4A (coordinate for the first peak: 605,306,311 bp), 5A (516,960,980 bp), and 7D (542,590,594 bp) and ∼100 Mb regions on the chromosomes 6B (123,972,511 bp) and 7A (75,769,595 bp). Although we were expecting a higher G4 density at GC rich regions as G4 motifs are mainly composed of G bases, we did not observe a clear correlation between GC content and G4 distribution, as there are regions with low GC content that are enriched for G4 motifs; *i.e.*, ∼100 Mb on chromosome 7A.

We also compared the number of G4 motifs across individual chromosomes and within high confidence gene models (Table 1). Here, we indeed observed a non-uniform distribution of G4 motifs along the chromosomes: higher G4 density (93 G4s per Mb) in genic regions of high confidence gene models as opposed to the G4 density of 76 G4s per Mb in whole chromosomes (Table 1). Of 1,071,813 G4 motifs in all the chromosomes, 34,370 of them were within the gene body of high confidence gene models (note that total length of the high confidence gene models was ∼370 Kb as opposed to ∼14 Mb for whole chromosomes). If G4 motifs were distributed across chromosome fairly similarly to genic regions, we would expect 1,306,599 motifs across chromosomes, but this is not the case. Additionally, when low confidence gene models were included together with high confidence gene models, a total of 77,256 G4 motifs (7.2% of total) were found within the 1 kb upstream and genic regions, totaling to 92 G4s per Mb around genic regions. Overall, our results reveal a significant enrichment and a non-random distribution of G4s around genic regions based on two-sample *t*-test (p-value = 6.62E-21).

**Figure 3:**
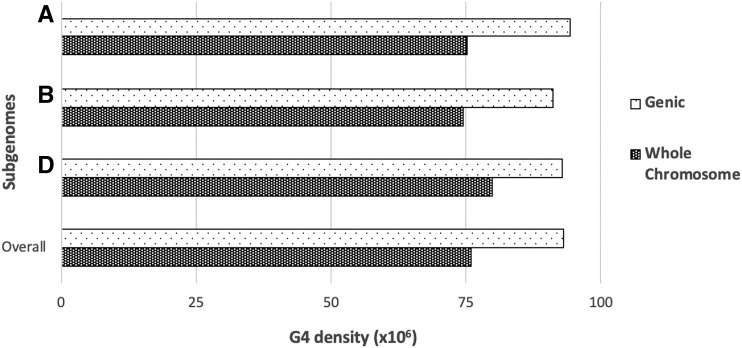
Distribution of G4 motifs over the subgenomes of hexaploid wheat. Dotted bars show the G4 density in genic regions and solid bars show the G4 density over subgenomes. G4 density was calculated as the number of G4 motifs per bp.

The hexaploid wheat genome evolved from hybridizations of three subgenomes, A, B, and D (Dvorák *et al.* 1993; Van de Peer *et al.* 2009; Jia *et al.* 2013; Marcussen *et al.* 2014). Each subgenome contributes equally to the gene content of wheat, although genome size varies with the D subgenome being the smallest followed by the A and B subgenomes (Appels *et al.* 2018). Interestingly, the highest motif density was observed in the chromosomes of D subgenome, 5D and 6D, whereas the lowest in that of B subgenomes, 4B and 2B (Table 1). In this study, we focused on the G4 motifs in each wheat subgenomes. Although G4 densities in genic regions are high in all subgenomes compared to the whole chromosome (Figure 3), subgenome-specific differences exist. For example, the density of G4 motifs across whole chromosomes were the highest in the D subgenome, and lower and similarly distributed in the A and B subgenomes (Figure 3). The higher density of the G4 motifs across whole chromosomes in the D subgenome is possibly due to the small genome size of the D subgenome. Our results show that genic regions are highly enriched by G4 motifs. The D subgenome contains similar number of gene models as the A and B subgenomes; however, the D subgenome size is ∼30% smaller than the B subgenome. Therefore, the increased G4 density across whole chromosomes in the D subgenome is likely associated with the small size of the D subgenome.

### G4 motif content in gene homeolog triads

Since G4 structures might interfere with the regulation of gene transcription, we evaluated and compared the distribution of G4 motifs in wheat triads with the relative expression of gene homeolog triads observed in a previous study. We retrieved a total 18,407 triads with exactly one copy of the homeolog genes present in the A, B, and D subgenomes (with the 1:1:1 correspondence) from the syntenic gene pairs that are available at wheat-URGI annotations. Our results show that most (58.1%) of the gene triads contains G4 motifs within the gene body and/or 1kb upstream of at least one homeolog (Figure S2). Although most of the triads contains G4 motifs, only 24% of the triads contains G4 motifs in all the homeologs in the three subgenomes. However, not all the homeologs contain G4 motifs in all the subgenomes and there are cases where G4 motifs are present or absent in a single homeolog with respect to the other two. For example, G4 motifs are present only in the A subgenome homeologs for 1,281 of the triads (with a G4 correspondence of 1:0:0) and absent only in the A subgenome homeologs for 839 of the triads (with a G4 correspondence of 0:1:1). These cases were defined as the A subgenome specific absence/presence of G4 motifs.

Overall, %34 of the gene triads showed such homeolog-specific absence/presence of G4 motifs among the A, B, and D subgenomes. We found that the absence/presence of G4 motifs in the D subgenome homeologs (1,951) is subtly smaller than the A (2,120) and the B (2,233) subgenomes. This homeolog-specific bias might have an effect on the relative gene expression of the homeologs since these G4 structures might interfere with the transcription of the genes. This finding is consistent with the relative expression of gene homeolog triads observed in a previous study (Ramírez-González *et al.* 2018) where authors observed homeolog-specific expression patterns for ∼30% of wheat triads with a slight increase in the relative expression of the homeologs in the D subgenome. Additionally, authors reported that single-homeolog suppression of the triads is more frequent than single-homeolog dominance. Supporting repressive effect of G4 motifs on gene expression, we observed increased cases of homeolog-specific presence (20%) rather than the absence (14%) of G4 motifs, further suggesting the reverse correlation of the absence/presence of G4 motifs with the homeolog specific expression of triads.

### G4 motifs are unevenly distributed around genes

**Figure 4:**
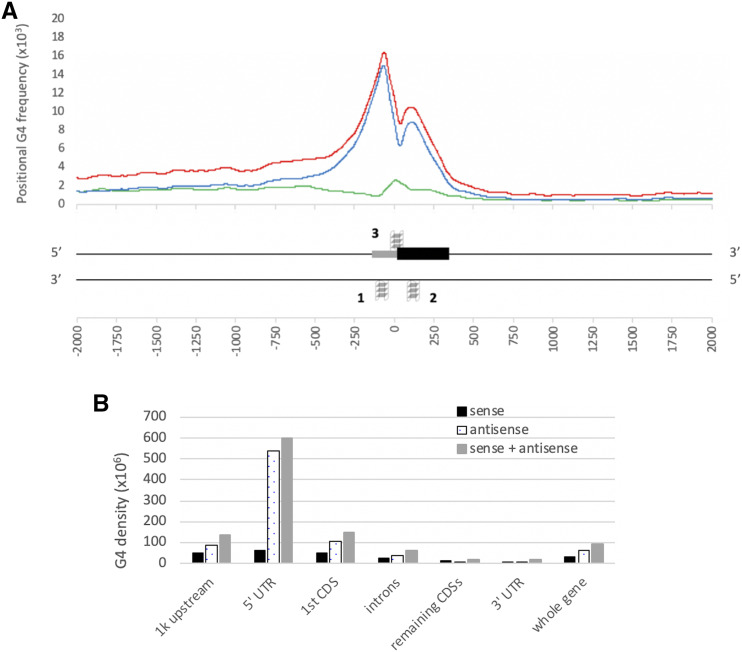
Distribution and breakdown of G4 motifs over gene structural elements in wheat genome. A: The 2,000 bp region comprising upstream and downstream of the start codon. The positional G4 frequency at specific positions were plotted as the average number of bases overlapped with a G4 motif per gene. Results were averaged over 100 bp window and centered at start codon positions. Plots are for G4 motifs on the sense (black), the antisense (dotted lines), and both strands (gray). G4 structures around genic regions on both strands are represented according to the peaks in the plot. Only the first CDS (black box) and the 5′ UTR (gray box) are shown. Peaks on antisense strand are labeled as 1 and 2, and peak on sense strand as 3. B: Histogram of G4 density distribution across gene structural elements. G4 densities were plotted as the average number of G4s within gene structural elements as follows: 1,000 bp upstream of 5′ UTR, 5′ UTR, 1^st^ CDS, remaining CDSs, introns, 3′ UTR, and the whole gene. Overall G4 density was shown in gray bars, G4 density on the antisense strand in dotted bars, and G4 density on the sense strand in black bars.

Driven by the evidence of G4 enrichment in transcription- and translation-specific regions in humans and plants including Arabidopsis and rice (Mullen *et al.* 2010; Takahashi *et al.* 2012; Kopec and Karlowski 2019), we examined the G4 enrichment pattern within the 2,000 bp of the start codons for the high confidence gene models in wheat. Our results suggest a nonuniform distribution of G4 motifs around and within genic regions in wheat (Figure 4). Using the positional G4 frequency plot centered at the gene start codon, we found that the highest enrichment is in the close proximity to the start codon (Figure 4A). We plotted the distribution of G4 motifs at transcript and gene levels (Figure S3). We did not observe a biased distribution: the distribution of G4 motifs were nearly identical between high confidence transcripts and gene models. G4 frequency was greater than 5 bases per Kb within -500 and +300 bp from the start codon, which was herein defined as the G4-rich region.

Intriguingly, we noted a sharp decrease in the overall positional G4 frequency at the start codon, flanked by two peaks. The first and the highest peak region between -300 to 0 bp covers the 5′ UTR region and about 100 bp upstream of the TSS sites. The second peak region between 40 and 240 bp covers the first CDS partially since the average sequence length of the first CDS for the genes with more than a single exon is ∼330 bp (∼470 bp on average overall, because single exon transcripts tend to have longer CDSs). Even though highly dependent on the length of the gene structural elements (spanning the 5′ UTR and the CDSs), these peaks correspond to the middle of the TSS and the first CDS regions for most wheat genes. In addition, we observed that G4s on the sense strand peak on the start codon though its magnitude is low compared to the whole gene. In fact, we observed two very close peaks on the sense strand of the start codon, but these peaks were merged into a single peak when a sliding window size of 100 bp is used (Figure S1A). We also analyzed 5,000 bp upstream and downstream regions, but we don’t see any other discernable G4 peaks (Figure S1A).

**Figure 5:**
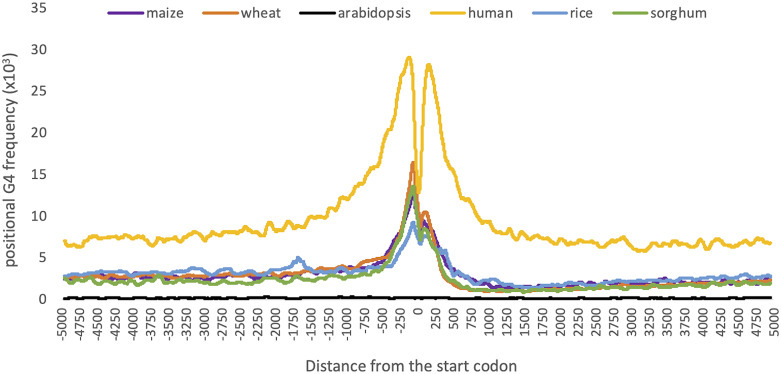
Distribution of G4 motifs across the 10,000 bp region centered at the gene start codon. Positional G4 frequencies were calculated as the number of G4 motifs at a given position, normalized by the number of genes. Moving averages were calculated with a window size of 99 bp. Species were colored yellow for human, orange for wheat, purple for maize, green for sorghum, blue for rice, and black for Arabidopsis.

Although G4 motifs are highly enriched between -500 and +300bp from the start codon, we observed an abundance of G4 motifs in the upstream of the genic regions when compared to their downstream (Figure 4A). Between +300 and +1,000 bp from the start codon, where the introns, secondary exons, and/or 3′ UTR are located, there was a significant decrease in the number of G4s. On the other hand, we noticed a prevalence of G4 motifs between -1,000 and -500 bp from the start codon (Figure 4A). A density graph in the masked maize genome suggested the opposite in the distribution of G4 motifs in the vicinity of the TSS sites in maize (Andorf *et al.*, 2014), where although -100 and +300bp from TSS sites were the most enriched for G4 motifs, they were more abundant in genic regions and the downstream of TSS sites compared to their upstream. We hypothesized that the discrepancy observed within the upstream and downstream of the TSS site originated from repeat masking of the genome. Upstream of the TSS site corresponds to the non-genic regions whereas downstream of TSS regions, between +300 to +1,000 bp from the TSS site, mostly corresponds to genic regions covering introns, secondary exons, and 3′UTRs. Because repetitive elements are more prevalent in intergenic regions rather than genic regions, repeat masking of maize genome might have concealed the G4 motifs present within the upstream regions of TSS. Indeed, our results show a higher abundance of G4 motifs within the upstream of genic regions in the unmasked genome of maize, similar to wheat and two other monocots (Figure 5).

Additionally, previous studies reported that G4 motifs were the most abundant in the CDS regions in Arabidopsis, and in the both 5′ UTR and CDS regions in rice (Kopec and Karlowski 2019). In contrast, G4 enrichment patterns differed in the human genome where G4 motifs were the most abundant in the 5′ UTR with at least 4 times more prominent than in the CDS regions (Kopec and Karlowski 2019), similar to what we observe here for the wheat genome. These results imply that there are minor differences in the distribution of G4 motifs over genic regions; however, the predominance of G4s in the close proximity of TSS sites and first exons were conserved in many species, thus implicating G-quadruplexes in regulating transcription and translation.

We examined the G4 densities in each gene structural elements separately (Figure 4B). In the 5′ UTR, exon, intron, and 3′ UTR regions, the enrichment profiles of G4s indicated that the 5′ UTR is the most prominent region for G4s (Figure 4B). The G4 density in the 5′ UTR was nearly 5 times higher than that of the whole gene. The next highest motif density was observed in the first CDSs which was only one fourth of that of the 5′ UTR. G4 structures within the 5′ UTR and CDS regions were previously suggested to be translation inhibitors (Bugaut and Balasubramanian 2012; Nie *et al.* 2015). For example, inhibition of unwinding of G4 structures within the 5′ UTR of an oncogene were resulted in the translational inhibition of these mRNAs (Wolfe *et al.* 2014).

Furthermore, our results revealed that G4s were more abundant in gene upstream regions than genic regions, possibly due to large CDS, intron, and 3′ UTR regions lowering G4 density. The average G4 density within the 1kb upstream of genic regions was nearly the same with that of the first CDS (Figure 4). This region mostly covers the promoter region where regulatory elements are located and is required for the transcription activation. G4 structures within promoter regions were shown to either inhibit transcription (Cogoi *et al.* 2014) **or** enhance transcription by recruitment of regulatory elements to the target site (David *et al.* 2016). Therefore, enrichment of G4 motifs within the regulatory regions suggest that G4s may play an important role in regulating gene expression, transcription, and translation. Although we provide the first insights into the mechanism underlying G4 functions in wheat here, further studies are required to understand how plant G4s play particular species-specific roles in cells.

### G4 enrichment in the vicinity of the start codon in humans and plants

Although G4s are enriched in the vicinity of the start codon in both human and plants, their magnitude and distribution show species-specific differences. Most notably, positional G4 frequencies were significantly higher in the human genome and significantly lower in Arabidopsis compared to monocot plants (Figure 5).

Among monocots, G4 density distributions over genic regions and upstream were relatively conserved (Figure 5). All four monocot plants shared the two peaks on the upstream and downstream of the start codon. Only rice exhibited a unique distribution pattern as to where two other peaks observed: one at the ∼1,750 bp upstream and another at the ∼300 bp downstream of the start codon (Figure 5). These results imply that there are minor species-specific differences in the distribution of G4 motifs over genic regions; however, the predominance of G4 motifs in the close proximity of TSS sites and first exons were conserved in many species.

These distribution graphs show differences based on selecting around which the plot is centered. Selecting either TSS or 5′ UTR start sites as center, the two peaks in the distribution graph cannot be observed, possibly due to the incompleteness of 5′ UTR annotations for many genes in reference genomes (data not shown). However, CDS sites are more prominently annotated for most of the genes. Therefore, we centered our graphs on the start codon/the first CDS start site. Surprisingly, by changing the center position, we observed the sharp decrease in the G4 frequency on the first CDS start site.

### G4 motifs on the sense and antisense strands are similar in number but differ in distribution

Intriguingly, the wheat reference genome contains similar numbers of G4 motifs on the both antisense and sense strands such that the difference is less than 1%. However, the majority of G4 motifs tend to be on the antisense strand of the genic regions (Figure 4A). The only enrichment peak of G4 motifs on the sense strand was identified around the start codon (Figure 4A). Interestingly, G4 densities were similar on the sense and antisense strands for the genic regions, and were only loosely associated to regulatory elements, *i.e.*, introns, CDSs other than the first one, and 3′ UTR regions (Figure 4B). It is also important to note the sudden decrease in the positional G4 frequency around the start codon flanked by two peaks. Based on the distribution of G4 motifs on sense and antisense strands, Figure 4A shows the most enriched regions around genic regions.

### Enriched gene ontology (GO) terms associated with genes containing G4 motifs

Structural elements within gene body and in the upstream of genes where most gene regulatory elements are located can interrupt proper functioning of genes. Therefore, we selected the genes containing G4 motifs within their gene body and 1,000 bp upstream and analyzed their gene ontology (GO) term assignments to understand what gene functions may be regulated by G4s. Of 105,200 high confidence gene models, 31,692 (30.13%) contain at least one G4 motif within gene body and its upstream. Among the genes containing G4 motifs, only 20,987 (66.22%) were in fact assigned at least one GO term. Most of these genes were involved in metabolic and cellular processes in the Biological Process category, and binding and catalytic activities in the Molecular Function category (File S2).

**Figure 6:**
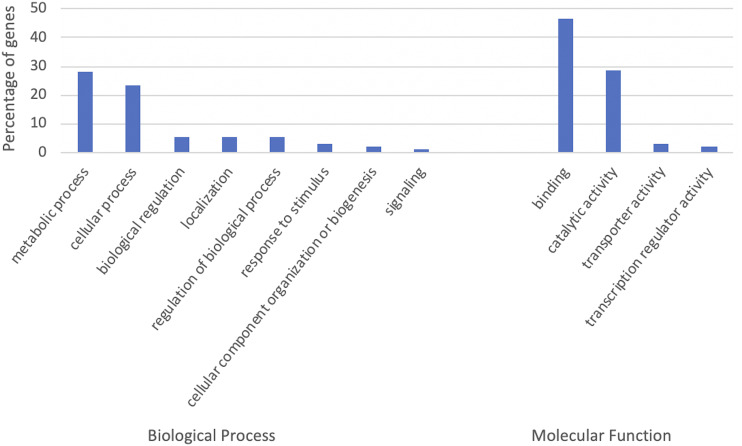
Biological processes and molecular functions enriched in G4 motif containing genes. The x axis shows the GO terms at level 2 in the GO hierarchy, and the y axis the percentage of genes among G4 motif-containing genes with at least one GO term. GO terms enriched in more than 1% of genes were shown.

We performed GO term enrichment analyses for the genes containing G4 motifs using the BiNGO plugin for the Cytoscape visualization tool (Maere *et al.* 2005). 19,603 (61.85%) of the genes were associated with enriched GO terms. Genes with enriched GO terms were plotted in Figure 6 for the Biological Process and Molecular Function categories at level 2 in the GO hierarchy. The GO enrichment analysis revealed that the G4 motif-containing genes are enriched in 333 GO terms in biological processes, such as in regulation of gene expression (GO:0010468), regulation of transcription (GO:0006355, GO:0006357, and GO:0045449), histone methylation (GO:0016571), and chromatin modification (GO:0016568 and GO:0016569). In addition, 210 GO terms in the molecular function category, which are mostly associated with either binding or catalytic activity, are enriched in the G4 motif-containing genes, such as hydrolase activity (GO:0016818, GO:0016817, GO:0016787, GO:0016790, and GO:0016820) and histone methyltransferase activity (GO:0042054).

We performed a comparison of GO terms at both gene and transcript levels. At the transcript level, of 130,745 high confidence transcripts, 45,483 (34.79%) contain at least one G4 motif within gene body and its upstream whereas only 30,760 (67.63%) were assigned at least one GO term. Our results showed that GO terms assigned to high confidence transcripts and genes were similar with only slight increase in the percentage of the transcripts assigned to binding at Biological Process category (46.28% of the transcripts as opposed to 43.98% of the genes). However, enriched GO terms varied between gene and transcript level analysis when enrichment calculated for genes against all gene annotations and transcripts against all transcript annotations. Enriched GO terms that were significantly different between genes and transcripts were evaluated using WEGO 2.0 web tool (Ye *et al.* 2018) based on the Chi-square test of independence (p-value < 0.05) and are shown in Figure S4. Remaining GO terms were similar at both transcript and gene level analyses. Some GO terms were enriched only at transcript level analysis such as histone acetylation (GO:0016573) in the BP category and histone acetyltransferase activity (GO:0004402) in the MF category.

Takashi *et al.* (Takahashi *et al.*, 2012) also performed GO enrichment analyses for many species for the genes containing G4 motifs within genes and 500 bp flanking regions. Although their surveyed region was not exactly the same as in our study, similar to what we observed here, they showed that some GO terms such as GO:0006355 (regulation of transcription, DNA-dependent), GO:0016573 (histone acetylation), and hydrolase activity (GO:0016818) are enriched in the G4-rich genic regions in other species including human, *Oryza sativa*, and *Arabidopsis thaliana* (Takahashi *et al.* 2012). The presence of GO terms that are enriched in a wide range of species in similar patterns supports the idea of the functional conservation of the genes containing G4 motifs in regulating similar biological processes and performing similar molecular functions.

### Functional enrichment of genes containing G4 motifs in the vicinity of the start codon

Our results above showed enrichment of G4 motifs in the vicinity of start codon (Figure 4). In this section, we strictly limit our analysis to the peak regions, *i.e.*, two peaks on the antisense strand and one peak on the sense strand as shown in Figure 4A. The total length of the three peaks was smaller than 600 bp. Although this region was much smaller than the total of span of the genic region together with 1,000 bp upstream, 19,804 (44%) transcripts of 15,163 high confidence gene models contain at least one G4 motif within these peak regions, supporting the significance of this small region around the start codon. GO term enrichment of the transcripts containing G4 motifs within peak regions against all high confidence transcripts revealed that these transcripts were involved in several important biological processes including response to stimulus, localization, and cellular component organization or biogenesis, in addition to metabolic and cellular processes (File S3). GO term assignment at level 5 showed that these transcript with enriched GO terms for response to stimulus process include L-ascorbic acid binding (File S3). In addition to be an important antioxidant that fight against the oxidative stress induced by both biotic and abiotic factors (Conklin 2001), L-ascorbic acid level were associated with flowering time and senescence (Barth *et al.* 2006). Additionally, several ubiquitin processes were also uncovered as enriched GO terms for transcripts containing G4 motifs within the peaks. Overall, these results clearly show the spectrum of important biological processes that might be affected by G4 motifs.

We then focused on the transcripts enriched for G4 motifs in these peak regions. 1,847 of the gene models contain more than one G4 motif in different peak regions whereas only 29 gene models contained G4 motifs in all three peak regions (Figure S5). These results suggested that although one third of the gene transcripts have G4 motifs within 1kb upstream and genic regions, much fewer of them actually have G4s in all three regions simultaneously. This is not only because of the low abundance of G4 motifs on the sense strand but also because of the small surveyed region for Peak 3: 300 bp for Peak 1 and 200 bp for Peak 2, compared to only 100 bp for Peak 3 (Figure 4A). In addition to transcripts containing G4 motifs at different peak regions, some transcripts contain multiple G4 motifs in one peak region. At total, 3,555 transcripts contain more than one G4 motif within the peak regions. Functional annotations showed that the transcripts encoding Ankyrin repeat proteins, F-box proteins, RING superfamily proteins, together with several kinases and histone proteins were enriched for G4 motifs within the peak regions (File S3 when filtered by total number of G4s >1).

**Figure 7:**
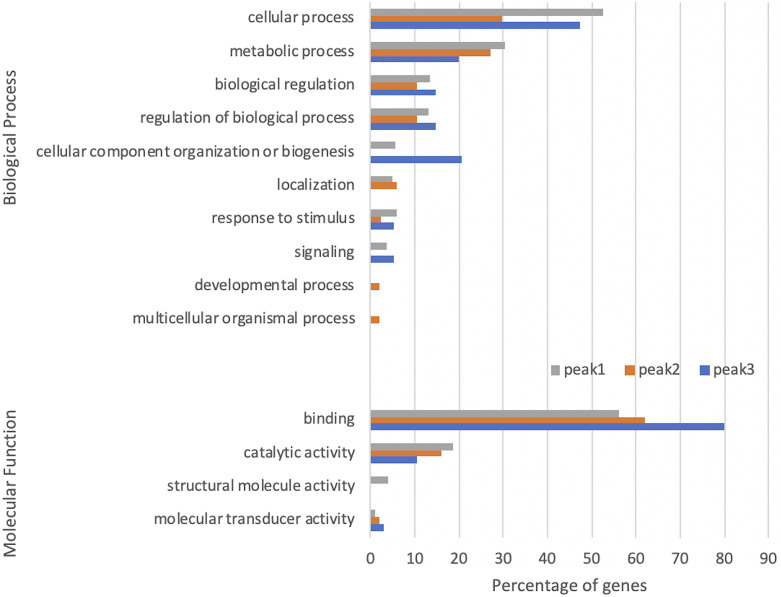
Peak-specific biological process and molecular function GO terms enriched in genes containing G4 motifs within G4-rich regions. The x axis shows the percentage of genes among G4 motif containing genes with enriched GO terms, and the y axis s the GO terms at level 2. Total numbers of genes with enriched GO terms for the peaks 1, 2, and 3 are 1203, 538, and 135 respectively. GO terms enriched in more than 1% of genes were shown. The peak numbering is shown in Figure 4A.

We evaluated further the functional enrichment in the genes containing G4 motifs within the peak regions (Figure 7). The most distinguishable differences in the enriched GO terms were observed for that of Peak 2. Developmental process and multicellular organismal process terms in the BP category were only enriched in the genes containing G4 motifs on the antisense strand of the first CDSs (Peak 2). Localization term in the BP category, on the other hand, was enriched in both Peak 1 and Peak 2 whereas structural molecule activity terms in the MF category were only enriched in Peak 1. However, none of these terms enriched in Peak 3, suggesting dissimilar functional regulation for the G4s on the antisense strand. On the other hand, the enriched terms of cellular component organization or biogenesis in the BP category and binding in the MF category were most abundant for Peak 3. These results suggest that G4s may be involved in different functional mechanisms depending on the localization on the respective gene.

### Case study: G4 motifs in the regulators of wheat flowering time

As a case study, we analyzed the G4 motifs in the wheat genes that are previously characterized as the regulators of flowering time (Appels *et al.* 2018). Flowering time is mediated by the epistatic interaction between the VRN-1 and VRN-2 genes. The VRN-1 gene encodes the MADS transcription factor where its ortholog is the AP1 gene in Arabidopsis (Yan *et al.* 2006). The VRN-2 gene is a repressor of flowering in plants and is negatively regulated by the VRN-1 gene (Distelfeld *et al.* 2009). Therefore, a high expression of VRN-1 gene is required to repress VRN-1 for flowering to occur. Winter varieties require vernalization, *i.e.*, exposure to long period of cold to flower (Sharma *et al.* 2017) to sustain stable high expression levels of VRN-1 (Trevaskis *et al.* 2006). In the spring wheat lines, the VRN-1 gene is dominant and is independent of vernalization (Yan *et al.* 2006).

Although genes regulating flowering time have been studied extensively, only some regulatory sites and important elements were identified for the VRN genes in wheat. For example, previous studies had associated the dominant VRN-1 gene with mutations and deletions in the promoter regions (Yan *et al.* 2003). However, Fu *et al.* suggested that not only promoter regions but also a region of intron 1 contains regulatory sites that are important for the recognition of VRN-1 for the repression by VRN-2 (Fu *et al.* 2005). The 2.3 kb region between 1.2 and 4.0 kb from the intron 1 start was suggested as a “critical region” where regulatory elements are located (Fu *et al.* 2005).

To reveal some of the putative regulatory elements in the VRN-1 genes and to explore the potential regulatory mechanisms in flowering time, we analyzed G4 distribution among the MADS transcription factor encoding genes in wheat with a specific focus on the promoter and the intron 1 regions (Yan *et al.* 2003; Fu *et al.* 2005). Both the 5′ UTR and intron 1 start regions have been previously reported to be highly enriched regions for G4s in human and maize (Maizels and Gray 2013; Andorf *et al.* 2014).

**Figure 8:**
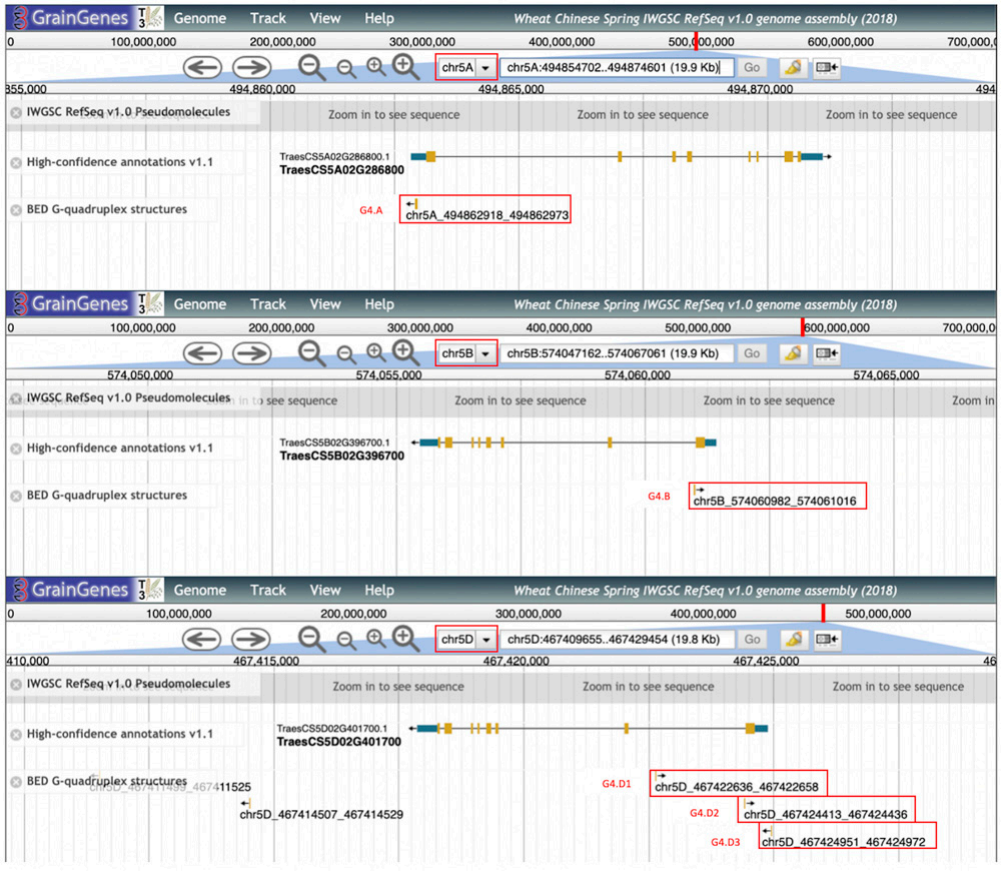
Examples of MADS genes overlapping with G4 motifs in the vicinity of start codons. Three windows from the GrainGenes genome browser for the IWGSC Chinese Spring wheat (Blake *et al.* 2016) were shown for chromosomes 5A, 5B, and 5D. Black arrows indicate direction of transcription (5′ to 3′ end). Gene elements are represented as yellow boxes (CDSs), blue boxes (UTRs), and black lines (introns). G4 structures intersecting with the genic regions were identified and indicated in red boxes.

We specifically examined a subset of the high confidence gene models that are putatively annotated by the IWGSC as the MADS genes that have functional orthologs in Arabidopsis and rice (Appels *et al.* 2018). A few examples from these gene clusters, together with the G4s in close proximity, were shown in Figure 8. In this group of genes, G4s are located mostly close to the gene start sites rather than to the 3′ UTR. For example, TraesCS5A02G286800 contains a G4 motif on the antisense of the 5′ UTR (shown as G4.A in Figure 8) whereas TraesCS5B02G396700 contains a G4 motif, on the antisense of the intron 1 start (G4.B).Some MADS genes contain more than one G4 motifs within their gene body and promoters. TraesCS5D02G401700, for example, contains three G4 motifs; one on the promoter (G4.D3), another on the start of intron 1 (G4.D2) and the last one within the 1.8 kb from the first intron start (G4.D1) (Figure 8). Interestingly, 1.8 kb from the first intron start site is within the “critical region” described by Fu *et al.* (Fu *et al.* 2005), supporting potential functional importance of G4 structures. Therefore, our G4 data provide previously uncharacterized regulatory elements in wheat that might affect known regulators of flowering response.

## Conclusions

G-quadruplexes, four stranded nucleic acid structures, were identified as important players in various biological process. Formation of these structures may block or hinder the accessibility of genomic regions by other regulatory proteins and elements. *In vivo* studies indeed showed the existence of special helicases to unwind G-quadruplex structures during transcription and inhibition of translation through stabilization of G-quadruplex structures by small ligands. Many studies have focused on the animal genomes and several important genes regulated by G-quadruplexes were identified. G-quadruplex structures have been studied as new therapeutic agents toward cancer therapy in humans (Asamitsu *et al.* 2019). However, only limited information is available for plant species. Understanding the role of these structures in plants will contribute to the plant biology and may lead future advances in agriculture.

In this study, we identified putative G quadruplex structures in wheat for the first time. Remarkably, one-third of high confidence gene models contain G4 motifs within their gene body and the 1,000 bp upstream region. Our results showed enrichment of G4 motifs specifically within the region covered -500 and +300 bp from the start codon, with two distinct peaks on the antisense strands of the both sides of the start codon, and a single peak in the sense strand around the start codon.

Genes containing G4 motifs within different peaks were associated with a range of biological processes, especially with structural molecular activity for the G4s on the antisense of the TSS, developmental process for the ones on the antisense of the first CDS, and cellular component organization or biogenesis for ones on the sense strand of the start codon. Outside the G4-rich region, G4 motifs were more abundant in the upstream of a gene than the genic regions. This enrichment profile over regulatory important regions imply potential functional importance of G4s in the regulation of transcription and translation.

We provided examples of G4 motifs nearby important genes such as the VRN-1 genes that regulate the flowering time in wheat. Our results identified the presence of G4 motifs within the critical regions and suggested potential regulatory roles in important biological processes. We also compared the genomic distribution of G4 motifs in wheat to that for five other species including human, Arabidopsis, maize, rice, and sorghum. Similarities in the G4 distributions among wide range of species indicate a widespread conservation and suggest a functional relevance.
